# Inhibition of thalamic relay nuclei scales the aperiodic and alpha band oscillations associated with arousal during naturalistic stimulus viewing

**DOI:** 10.1162/imag_a_00451

**Published:** 2025-01-23

**Authors:** Ritu M. Borah, Anagh Pathak, Arpan Banerjee

**Affiliations:** National Brain Research Centre, Gurgaon, India

**Keywords:** arousal, valence, aperiodic, periodic, corticothalamic model, neural field

## Abstract

Existing psychological theories posit valence and arousal as two dimensions along which subjective emotions’ states are represented. The present study sought to determine whether the emotional ratings projected along the valence–arousal axis during naturalistic stimulus viewing are further mapped in the neurodynamical attributes observed from noninvasive electroencephalography (EEG) in humans and their potential biophysical causes. While several studies have explored the periodic features of EEG dynamics of emotion processing, very few studies have previously explored the aperiodic (1/f) components vis-à-vis emotion arousal. Recent signal processing developments have established that segregating EEG time series into aperiodic and periodic components provides fundamental insights underlying neural computations, specifically excitatory–inhibitory balance. In this study, we identified that there is a significant increase in exponent and offset of aperiodic background during arousal. In contrast, there were no discernible variations between the aperiodic components during the differential valence scenarios. Reduction in periodic alpha power was observed with high arousal in line with previous studies. Finally, implementation of a biophysically realistic corticothalamic model of neural field activity allowed us to mechanistically explain that both the empirical observations of heightened arousal represented as higher slope of aperiodic background and decrease in power of the periodic alpha oscillations emerge from an increased inhibitory influence to relay nuclei of the thalamus.

## 1 Introduction

Emotions play a pivotal role in modulating human perception and action (Zadra & Clore, 2011), attention (Carretié et al., 2001;Dolcos et al., 2020), enhancing memory (Dunsmoor et al., 2019;Tyng et al., 2017), and social and economic decision making (Eijlers et al., 2019). Hence, understanding the neurophysiological mechanisms underlying our emotional experiences offers a unique perspective to investigate various cognitive functions. A well-established conceptual framework identifies two dimensions—valence and arousal, along which emotional states can be quantified (Posner et al., 2005;Russell, 1980). Valence pertains to the spectrum of unpleasant to pleasant emotional experiences, while arousal relates to the degree of excitement associated with an emotional state. Several studies have employed electroencephalogram (EEG) paradigms involving stimuli such as facial expressions, music, and virtual reality experiences to induce emotions and explore their neural correlates (Costa et al., 2006;Daly et al., 2014;De Cesarei & Codispoti, 2011;Hofmann et al., 2021). For instance, a decrease in alpha power has been consistently associated with high arousal states (Hofmann et al., 2021;Kim et al., 2021;Schubring & Schupp, 2021). However, it is worth noting that a contrary finding of increase in alpha power correlated with increased arousal also exists (Aftanas et al., 2004;Uusberg et al., 2013). Alpha power decrease is also observed with negative emotions, for example, fear (Eijlers et al., 2019). Additionally, beta power reductions have been linked to increased arousal (Kim et al., 2021;Schubring & Schupp, 2021), while frontal midline theta (4–7 Hz) activity has been correlated with pleasant stimuli (Sammler et al., 2007). While these studies highlight the significance of rhythmic oscillations in understanding the neural underpinnings of emotion, an explanation of the disparate findings related to alpha increase or decrease remains elusive.

A major thrust in neural signal processing has recently shifted to the understanding of both rhythmic and arrhythmic (nonoscillatory or 1/f) components (He, 2014;He et al., 2010), the latter being hypothesized to confound the measurement of true neural oscillations (Gyurkovics et al., 2021;Voytek et al., 2015). Intriguingly, researchers have also proposed that the 1/f activity is a hallmark of criticality (Buzsáki, 2006)—the neurobiological state of homeostasis among excitatory and inhibitory populations (Donoghue et al., 2020;Gao, 2016;Voytek et al., 2015) maintained in a putative brain region to process incoming stimuli. Supporting this possibility, emerging evidence suggests that arrhythmic components also hold relevance as correlates of cognitive processes, for example, predicting cognitive processing speed (Ostlund et al., 2021;Ouyang et al., 2020), charting the lifespan trajectories of cognitive processing (Thuwal et al., 2021), thus necessitating a comprehensive approach to their analysis (Colombo et al., 2019;Lendner et al., 2020;Voytek et al., 2015). Finally, the accurate characterization of the arrhythmic activity can also reconcile the disparate observations, such as alpha decrease/increase in different cohorts (Gyurkovics et al., 2021). Consequently, it is imperative to investigate the role of aperiodic brain dynamics in shaping emotional states—an area that has thus far remained scantly explored in the field of emotion research.

From a rigorous neurobiological perspective, numerous studies have illuminated the intricate involvement of subcortical regions—the thalamus, amygdala, and various sensory and frontal cortical regions—in processes related to emotional arousal and valence processing (Karjalainen et al., 2019). Notably, fMRI investigations consistently reveal a positive correlation between amygdala activation and emotionally arousing stimuli, irrespective of their valence (Lin et al., 2020) as well as existence of multitasking core systems that process emotion (Barrett, 1998,^2017^). Interestingly, excitatory connections from the amygdala to thalamic reticular nuclei, serving as an attentional regulatory mechanism, were identified in primates (Barbas et al., 2011).

In this paper, we employ a neural mass model to study the dynamic interactions between the thalamus and cortex, particularly in relation to emotional arousal. This model, first developed byRobinson et al. (1997,^2003^), has been applied successfully to various brain functions, including arousal, epileptic seizures, the sleep–wake cycle (Rennie et al., 2002), and chronic brain injury treated with Zolpidem (Victor et al., 2011). Considering these previous applications, we hypothesized that this model could offer valuable insights into the complex aperiodic dynamics underlying emotional arousal. Most importantly, this model can generate both aperiodic and periodic EEG spectra, making it a powerful tool for simulating and understanding brain activity. We specifically hypothesized that increased emotional arousal modulates the thalamic reticular nuclei, thereby facilitating enhanced inhibitory coupling with the relay nuclei which in turn feeds back to cortical population and governs EEG 1/f spectra.

The DEAP dataset is widely used for emotion recognition studies. It consists of physiological data, including EEG, EMG, and other biometric signals, collected from 32 participants while they watched music videos designed to evoke emotional responses. The dataset includes ratings of arousal, valence, liking, and dominance, allowing for a thorough analysis of emotional states (Koelstra et al., 2012). Numerous studies have utilized the DEAP dataset to develop and refine emotion recognition systems. The works have focused on using machine learning algorithms, such as support vector machines (SVMs) and deep learning models, to classify emotional states based on EEG signals (e.g.Chen et al., 2019;Jirayucharoensak et al., 2014). These studies have shown that EEG data are particularly effective in distinguishing between high–low arousal and valence, providing a strong foundation for exploring emotional dimensions.

In the context of our study, the DEAP dataset provides an essential basis for investigating both aperiodic and periodic neural activity within the emotional dimensions of arousal and valence. Our main objectives are twofold: first, to characterize EEG from the DEAP (Data for Emotional Analysis using Physiological Signals) database (Koelstra et al., 2012) into aperiodic and periodic components and to characterize whether they represent the dimensions of valence and arousal, and second, to explore the systems-level interactions that guide the empirical observed representations through the corticothalamic neural field model.

## 2 Methods

### 2.1 Dataset and experimental design

We utilized the open-source affective Database for Emotion Analysis using Physiological signals (DEAP). The dataset includes EEG recordings (at a sampling rate of 512 Hz, using Biosemi Active Two system) and physiological signals such as blood volume pressure, respiration rate, and temperature. The data were collected from 32 participants, with an equal distribution of male (16) and female (16) participants. The participants’ mean age was 26.9 years, ranging from 19 to 37 years.

**Fig. 1.**
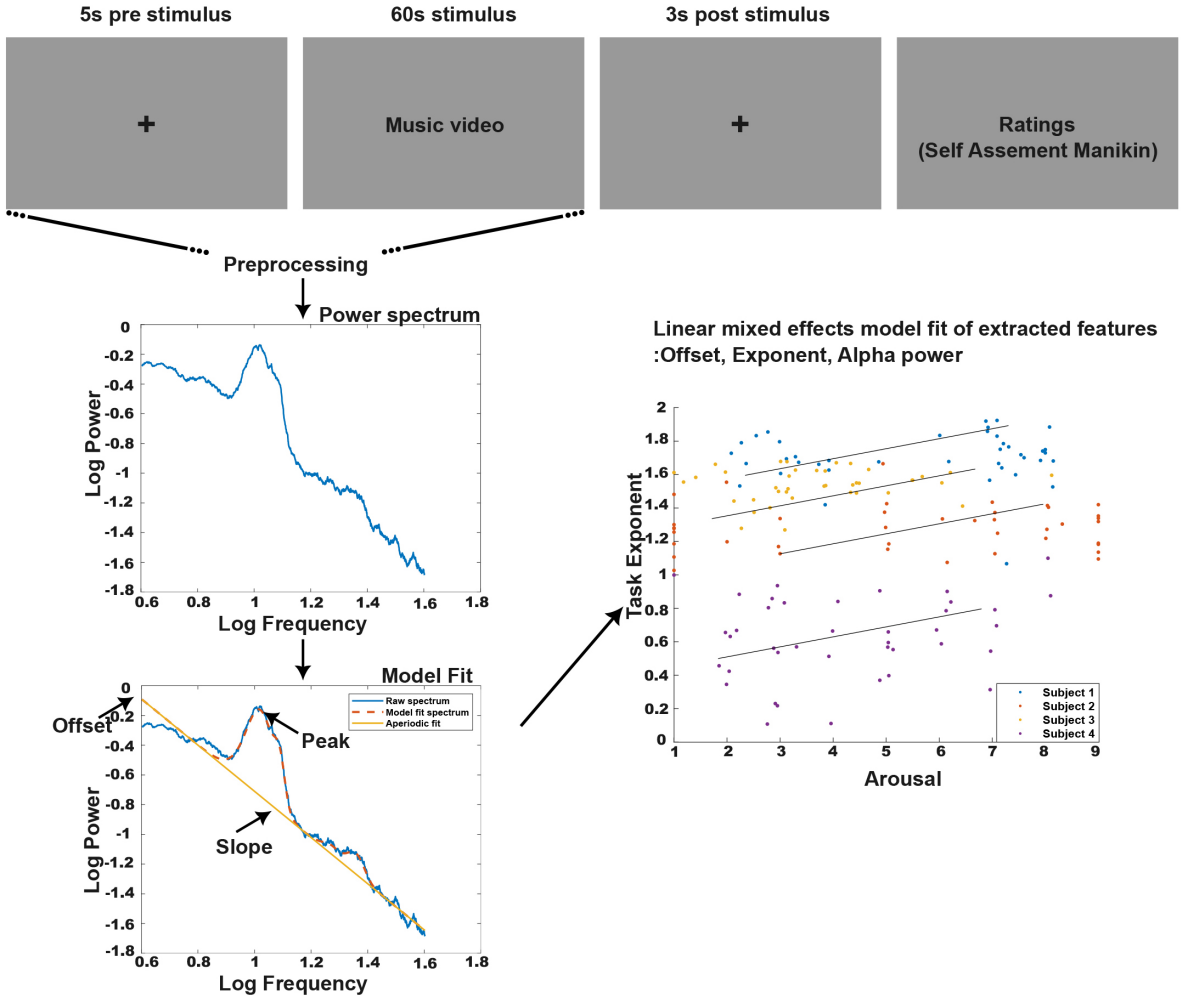
Representation of the stimulus presentation and methodological workflow. Each trial consists of a 5-s prestimulus fixation, followed by a 60-s music video, and ends with a 3-s fixation window. After each trial, participants rate Arousal and Valence; additional ratings for Dominance, Liking, and Familiarity were collected but are not included in the current study (seeSupplementary Fig. S1(Koelstra et al., 2012)). The methodological process includes power spectral analysis applied to the EEG data, followed by FOOOF to separate aperiodic and periodic components. Extracted features, such as offset, exponent, and power within specific bands, are then subjected to a linear mixed model analysis to investigate their relationship with the emotional ratings.

During the experiment (Fig. 1), each participant watched 40 different 60-s music videos. The presentation of the videos was separated by a fixed intertrial interval of 5 s. After viewing each video, there was a 3-s fixation window, and then the participants were asked to rate their emotional experiences using continuous 1–9 Likert scales for valence, arousal, dominance, and liking. Additionally, participants rated their familiarity with the videos on a Likert scale, 1–4. For the mentioned rating, relevant self-assessment manikins were displayed in the middle of the screen (Fig. 1). The participants rated their experience by moving the mouse below the manikins in a continuous scale. They were instructed to rate their valence from feeling of unhappy or sad to happy or joyful state. The arousal ranged from calm or bored to stimulated or excited. For the liking ratings, participants were instructed to express their preferences by indicating their levels of liking or disliking for the stimuli. Since we were interested in valence and arousal states, we only extracted only those two behavioral ratings for our study.

### 2.2 Preprocessing

We used EEGLAB for the preprocessing of the data. Initially, a bandpass filter with a range of 2–45 Hz (pop_eegfiltnew.m) was applied. Subsequently, bad channels were removed using cleanline function where a channel was removed if there is 5 s flatline, standard deviation of high-frequency signals is greater than 5 and the correlation to the nearest channel is less than 0.8. Epochs were extracted, spanning from -6 to 61 s from the onset of video stimuli, and then Independent Component Analysis (ICA) was performed (pop_runica). Artifacts were labeled using the default ICA label algorithm and a component was rejected if it was identified as eye, muscle above the 90% threshold. To establish a common reference, the data were re-referenced to the average (pop_reref).

The Multitaper method (mtspectrumc, CHRONUX) was employed to convert the time series data into the frequency domain. Prior to conducting the multitaper analysis, detrending was done (detrend.m). The power spectrum for each trial and channel was then estimated. To perform the multi-taper spectral analysis, we used a taper bandwidth of 0.6 Hz, corresponding to a time-bandwidth product of 36 (calculated as 60 s × 0.6 Hz) and applied 45 tapers. Additionally, the tapers bandwidth was kept consistent with the 5-s prestimulus fixation period, which utilized five tapers.

To assess for spatial topography-based effect of aperiodic and periodic parameters, the electrodes were categorized into prefrontal (AF3, AF4, FP1, FP2), frontal (F7, F3, Fz, F4, F8), central (C3, Cz, C4), centroparietal (CP1, CP5, CP6, CP2), parietal (P7, P3, Pz, P4, P8), parietooccipital (PO3, O1, Oz, O2, PO4), temporal (T8), and temporal (T7). PCA was performed on the time series of these clusters of electrodes and the first principal component transformed data were considered for further analysis on spatial distribution.

### 2.3 Estimation of aperiodic and periodic components

Fitting Oscillations and One Over F (FOOOF) toolbox in MATLAB was employed to parametrize the power spectra of the EEG data into periodic and aperiodic components (Donoghue et al., 2020). The FOOOF algorithm involves several steps to fit and analyze the spectra. Initially, it fits an aperiodic component, which is represented as a straight line when plotted in log–log frequency and power space, unless a knee or break in the spectrum is predicted or specified. This fitted aperiodic component is then subtracted from the original raw spectra, resulting in residuals. Next, the periodic and noise components are estimated based on these residuals. To identify peaks in the spectrum, a Gaussian function is fitted to the residual spectrum, and a peak is detected when the fitted function surpasses the noise threshold. The process of fitting the Gaussian is performed iteratively, depending on either reaching the maximum peak detection limit set or when the next Gaussian falls below the noise threshold. The final raw spectrum is obtained by fitting the Gaussian function(s) along with the aperiodic component, yielding a comprehensive representation of the entire spectrum. This allows for the separation and extraction of rhythmic and arrhythmic components.

In the modeling process, the power spectrum (PSD) was defined as the mix of Gaussian and Lorentzian function as inEquation (1)

$$PSD=L+\sum_{n=0}^{N}G_{n}$$ (1)

The Gaussian ($G_{n}$) is defined as function of peak power (a) in$log_{10}\left(power\right)$, central frequency (c), standard deviation (ω), and vector of input frequencies (F) as follows:

$$G_{n}=a\times exp\left(\frac{-{\left(F-c\right)}^{2}}{2}\omega^{2}\right)$$ (2)

The Lorentzian (L) giving the aperiodic fit is explained by the broadband offset (b), exponent (X), and knee parameter (κ) which determine whether the bend or break in the slope of the power spectrum

$$L=b-log\left(\kappa +F^{X}\right)$$ (3)

For the model fit, following parameters were applied. The frequency range was set to [4 40]Hz, the maximum number of peaks allowed was 5 for the 60-s stimulus fit and 5 for the 5-s prestimulus fit. The minimum peak amplitude was required to exceed the aperiodic fit, set at 0.0. The aperiodic mode was fixed, and the peak width limits were restricted to [0.5 12]Hz. Additionally, a peak threshold of 2.0 was used.

This procedure was employed on the power spectrum of the average of electrodes for each trial. The estimated exponent, offset, and peak alpha power were used for further analysis. In instances where multiple alpha peaks were detected, the one with the maximum power was selected for subsequent analysis. We subsequently fitted the model on the PCA reduced time series to investigate the topography of the spectral parameters.

### 2.4 Statistical paradigm

To investigate the effect of arousal, valence, and their underlying interaction effect on the spectral parameters (exponent, offset, alpha), we employed a linear mixed model with by-subject random intercept and slope (Barr, 2013). This inclusion of the by-subject random slope captures the subject-specific variations in arousal and valence, adjusting the predictions by a fixed value for each subject. The resulting formula is as follows:

Xis=β0+β1(Xpre)+β2(Ai)+β3(Vi)+β4(Ai×Vi)+S0 +[β2(Ai)+β3(Vi)]Sis+eis (4)

Xis=β0+β1(Xpre×Ec)+β5(Ec×Ai)+β6(Ec×Vi) +β4(Ai×Vi)+S0+[β2(Ai)+β3(Vi)]Sis+eis. (5)

Equation (4)is the general formula for linear model fit on average electrode spectral parameters (exponent, offset, alpha power), whileEquation (5)represents the cluster-based linear model fit.

Here,$\beta_{0}$represents the intercept of the linear model, reflecting the baseline level of the spectral parameters when all predictors are zero.$\beta_{1}$denotes the effect of the prestimulus spectral parameter ($X_{pre}$), which is used for baseline correction to account for preexisting differences before the task.$\beta_{2}$and$\beta_{3}$correspond to the fixed effects of arousal ($A_{i}$) and valence ($V_{i}$), respectively, while$\beta_{4}$represents the interaction between arousal and valence ($A_{i}\times V_{i}$).$S_{0}$is the random intercept at the subject level, capturing individual differences across subjects, and$S_{is}$represents the random slope, which accounts for how the effects of arousal and valence vary between subjects. Finally,$e_{is}$denotes the residual error, which captures the unexplained variability in the spectral parameters.

Here,$E_{c}$denotes the electrode cluster, allowing for the investigation of spatial variations across different brain regions. The term$\beta_{1}$($X_{pre}\times E_{c}$) introduces an interaction between the prestimulus spectral parameter and the electrode cluster, ensuring that baseline correction is applied specific to each region. Additionally,$\beta_{5}\left(E_{c}\times A_{i}\right)$and$\beta_{6}$($E_{c}\times V_{i}$) capture the interactions between the electrode cluster and arousal ($A_{i}$) and valence ($V_{i}$), respectively, reflecting how the effects of these emotional dimensions vary spatially across brain regions.

The interaction between arousal and valence$\beta_{4}$($A_{i}\times V_{i}$) remains consistent, while the random intercept ($S_{0}$) and random slopes ($S_{is}$) account for subject-specific variability, as described in the previous model. This approach allows us to examine not only the impact of arousal and valence on spectral parameters but also how these effects differ across various electrode clusters, adding a spatial dimension to the analysis.

Here, four subjects were removed due to insufficient number of trials containing central alpha peak frequency in the 8–12 Hz range. This variability could reflect individual differences in neural dynamics, however, to achieve a better model fit and minimize the amount of missing data, these individual variations were excluded from the analysis (Fig. S2). Further, from the remaining subjects, trials with negative exponent (positive slope) were removed. This negative exponent represents the trend of high power in high frequency, which could be reflective of noise.

### 2.5 Neural mass model of EEG dynamics

**Fig. 2.**
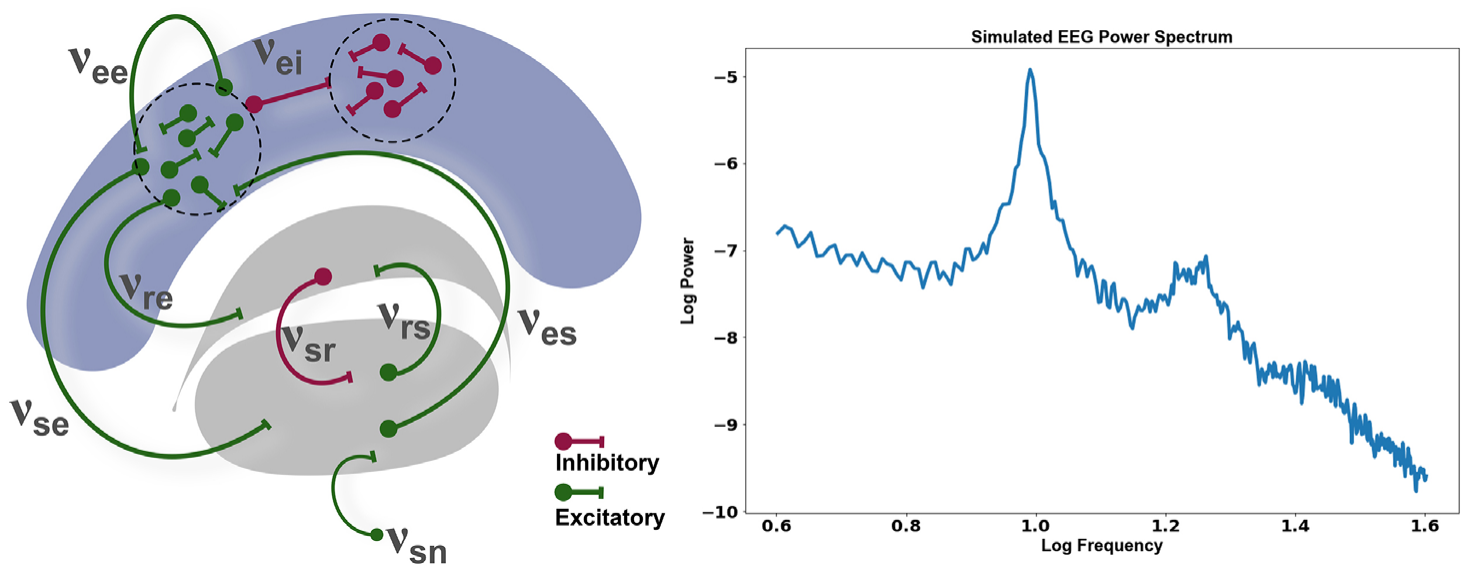
Schematic representation of the corticothalamic model showcasing feedforward and feedback loop between thalamus and cortex. Here,*e*represents excitatory,*i*represents inhibitory,*s*represents the relay nucleus of the thalamus, and*r*represents the reticular nucleus of the thalamus. φ denotes the presynaptic potential, and*V_ab_*represents the membrane potential, where the subscript*ab*indicates the specific interaction between neuronal populations (*a*,*b*∈*e*,*i*,*s*,*r*). The inset shows a simulated power spectrum using normative parameters (Table 1).

**Fig. 3.**
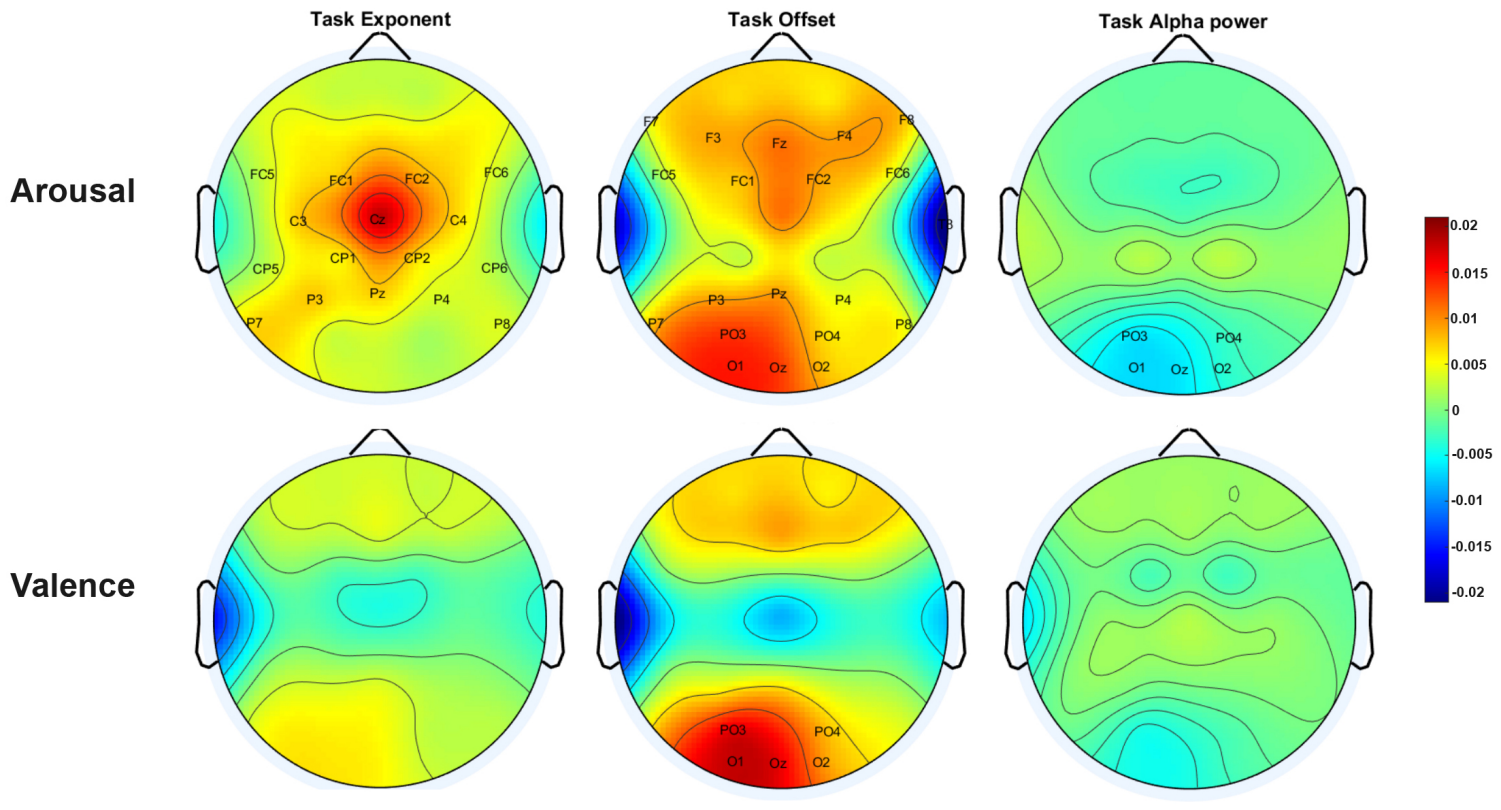
Topoplot of beta estimates from the linear mixed model (Equation 5), with electrode clusters displaying significant p-values labeled. Each channel within a cluster is reweighted by the PCA mixing matrix multiplied by the reported effect, emphasizing the spatial distribution of beta estimates across electrode sites. Arousal is represented in bilateral central sensors for task exponent. Both arousal and valence are represented only along task offset for left occipital sensors. Additionally, arousal modulated the task offset in frontal and frontocentral regions, indicating a global representation, and also modulated alpha power in parietooccipital sensors. The color bar denotes the beta estimates of the linear mixed model. The raw spectrum and FOOOF computed oscillatory and aperiodic components of spectrum are plotted inFig S3 a, b and c, respectively.

In order to arrive at a biophysical explanation for observed spectral changes in ongoing recordings as a function of valence and arousal, we employed a well-established neural mass modeling approach of EEG/MEG dynamics that incorporates the corticothalamic interactions (Fig. 2). The model consists of a recurrent population of cortical and thalamic neurons where the average dynamics of each population (neural mass) is described by coupled rate equations (Rennie et al., 2002;Robinson et al., 2003) (Fig. 3). The thalamus consists of reticular and relay populations. The excitatory relay cells convey ascending inputs to the cortex and are kept under inhibitory control by the reticular population which receives inputs from subcortical structures like amygdala (Barbas et al., 2011), cortical regions like prefrontal (Zikopoulos & Barbas, 2006). The cortex is represented as an excitatory–inhibitory mass that receives subcortical input from the relay cells and feeds back to the thalamic populations. Importantly, these interactions give rise to EEG power spectra that follow a$\frac{1}{f^{X}}$distribution, where the exponent (X) varies across frequency bands. For low frequencies,Xis typically close to zero, indicating a flatter spectrum, while at higher frequencies (10–50 Hz),Xapproaches 2, reflecting a steeper slope (Miller et al., 2009;Robinson et al., 2001). Beyond this range, there is a transition to an even steeper slope, which reflects the effects of dendritic filtering. This pattern suggests that the EEG is “close to critical” primarily near the zeroth and alpha modes, with higher frequencies being heavily damped. These dynamics are captured in our model, with corticothalamic interactions and synaptodendritic filtering, shaping the observed spectra (Robinson et al., 2001). All corticothalamic interactions are subject to a finite transmission delay which is kept as 40 ms throughout the article, keeping in line with previous studies. In the absence of overt stimuli, the relay populations are fed by noisy input. Each population in the model is characterized by its mean soma membrane potential (V), mean firing rate (Q), and the local presynaptic activity (ϕ). Differential equations are employed to specify the conversion of these quantities, mimicking the effects of neuronal gain and synaptodendritic filtering. The following second-order differential equation specifies how the mean membrane potential (V) of a population changes in response to input presynaptic activity (ϕ)

$$\frac{1}{\alpha \beta }\frac{\partial^{2}V_{j}}{\partial t^{2}}+\left(\frac{1}{\alpha }+\frac{1}{\beta }\right)\frac{\partial V_{j}}{\partial t}+1=\sum_{1}^{k}v_{jk}\phi_{k},$$ (6)

whereαandβare the inverse rise and decay times, respectively, of membrane potential generated by an input impulse. Here,$\nu_{jk}=N_{jk}s_{k}$is the synaptic coupling where$N_{jk}$is the mean number of synapses from neurons of typek(e.g.*e, i, s*) to typejand$s_{k}$is the strength of the response from neurons of typek. The resulting membrane potential (V) maps to the mean firing rate (Q) according to the following sigmoidal activation function

$$F\left(V\right)=\frac{Q_{max}}{1+exp\left(-\pi \left(V-\theta \right)/\sigma \sqrt{3}\right)},$$ (7)

where$Q_{max}$is the maximum firing rate,θis the mean firing threshold, andσis its standard deviation. Finally, the mean soma firing rate (Q) is converted to a resulting local presynaptic activity (ϕ) through the following damped wave equation

$$\left(\frac{1}{\gamma^{2}}\frac{\partial^{2}}{\partial t^{2}}+2\gamma^{2}\frac{\partial }{\partial t}+\gamma^{2}-v_{\;e}^{2}\nabla^{2}\right)\phi =Q,$$ (8)

$\gamma =\nu_{e}\text{​}/r_{j}$and$r_{j}$is the mean range of axons. Based onRobinson et al. (2001)considering intracortical connectivities proportional to the number of synapses, only excitatory population is considered. For normative parameter values (seeTable 1), the model mimics the dynamics of resting-state human EEG/MEG, with prominent peak in alpha (8–12 Hz) frequency band, and a distinct 1/f broadband slope. Note that here we let the Laplacian term$\nabla^{2}$inEquation (8)to be zero, implying that we study a global mode of the neural field equation. This approximation considerably simplifies the subsequent numerical integration while retaining a basic notion of spatial spread in the model (e.g. nonzero corticothalamic delays). The differential equations were numerically solved using the Euler–Murayama scheme (Python 3.4, NumPy, Windows 11) with an integration time step of 1 ms (seeSupplementary Textfor detailed equation for each level of connection).

**Table 1.** Baseline resting-state parameter values

Parameter	Resting-state value	Parameter description
θ	15 mV	Mean neuronal threshold
σ	6 mV	Threshold standard deviation
Q	250 $s^{-1}$	Maximum firing rate
γ	100 $s^{-1}$	Ratio of conduction velocity with mean range of axons
α	50 $s^{-1}$	Inverse decay time of membrane potential
β	240 $s^{-1}$	Inverse rise time of membrane potential
$t_{0}$	80 ms	Corticothalamic return time
$\nu_{ee}$	1.06 mVs	Excitatory to excitatory synaptic strength
$v_{ei}$	-1.8 mVs	Inhibitory to excitatory synaptic strength
$v_{sr}$	-0.845 mVs	Reticular to specific (relay) nucleus synaptic strength
$v_{sn}$	1.20 mVs	Nonspecific noise to specific nucleus synaptic strength
$v_{ℜ}$	0.91 mVs	Excitatory to reticular nucleus synaptic strength
$v_{rs}$	0.41 mVs	Specific to reticular nucleus synaptic strength
$\nu_{se}$	2.28 mVs	Excitatory to specific nucleus synaptic strength
$\nu_{es}$	2.20 mVs	Specific to excitatory nucleus synaptic strength

Adapted fromFreyer et al. (2011)andRobinson et al. (2001).

## 3 Results

### 3.1 Global exponent increase with arousal while no effect of global offset and alpha on arousal and valence

A linear mixed model was fitted using the restricted maximum likelihood method to analyze the effects of arousal, valence on the spectral parameters obtained during the music viewing and listening task, that is, task exponent, task offset, and peak global alpha power. As an additional variable of influence, prestimulus values of the respective parameters (prestimulus exponent, prestimulus offset, and prestimulus alpha) were also considered. The model also included random intercepts and slopes for arousal and valence (Equation 4) (Table 2). The fixed effects revealed significant contributions from the exponent during the prestimulus period (β = 0.2587, p < 0.001), with arousal showing a marginal effect (β = 0.0162, p = 0.0487) and no effect of valence. Interaction between arousal and valence also showed no significant impact. The random effects indicated considerable variability between subjects (σ = 0.2770). To correct for type-I error, Satterthwaite’s method was used with significant effects for prestimulus exponent and arousal (Table 2). There was a fixed effect of prestimulus offset (β = 0.2017, p < 0.001) while no main effect of arousal, valence, and their interaction (β = -0.001025, p = 0.448) was observed. Moreover, no fixed effects of prestimulus alpha, arousal, valence, and their interaction was found for task alpha power. However, there was considerable subject variability σ = 0.3507 and σ = 0.2669 for offset and alpha power, respectively.

**Table 2.** F-statistics with Satterthwaite adjusted p-value for the linear mixed model (Equation 4) applied on task exponent and task offset.

	Mean square	F-value	p-value
Dependent variable = task exponent			
Prestimulus exponent (1, 943.13)	4.5296	136.1105	<0.001
Arousal (1, 259.66)	0.1305	3.9217	0.0487
Valence (1, 352.85)	0.0088	0.2647	0.6072
Arousal × valence (1, 671.63)	0.0439	1.3118	0.2514

Dependent variable = task offset			
Prestimulus offset (1, 941.21)	2.78972	98.1800	<0.001
Arousal (1, 223.24)	0.04881	1.7176	0.1913
Valence (1, 323.25)	0.01307	0.4598	0.4982
Arousal × valence (1,603.8)	0.01636	0.5759	0.4482

### 3.2 Local variability of spectral parameters with arousal and valence

To further analyze the electrodes that significantly influence the modulation of spectral parameters associated with arousal and valence, we examined the statistical strength of these parameters locally across various brain regions. We grouped the electrodes into nine clusters based on their positions: central, centroparietal, parietal, parietooccipital, prefrontal, frontal, frontocentral, and bilateral temporal areas (Fig. 3). This clustering approach was employed to reduce the dimensionality for comparison.

We modeled the interaction of arousal, valence, and prestimulus spectral components with the electrode clusters as a function of task spectral components (Equation 5). This approach allowed us to assess the localized effects of emotional states. Our analysis revealed a significant effect of arousal on the task exponent at the central, centroparietal, frontocentral, and parietal regions. Similarly, we observed significant effects of arousal on task offset at the frontal, frontocentral, parietal, parietooccipital, and right temporal regions (Fig. 3). The task alpha power significantly decreased with arousal in the parietooccipital region, indicating changes in alpha wave activity related to arousal also reported in previous studies. Interestingly, valence had a significant effect on task offset at the frontal, parietooccipital, and left temporal regions.

To ensure the robustness of our findings, we applied the Holm method for multiple comparisons correction. The significant effects of arousal and valence that remained robust after applying this correction are reported inTable 3(referTable S1for full model result).

**Table 3.** Cluster-based interaction mixed model estimates with Holm adjusted p-value showing only significant interactions.

Factors	β	Standard error	Z-value	p-value
Dependent variable = task exponent				
Arousal×C	0.0345	0.0074	4.6330	<0.001***
Arousal×CP	0.0260	0.0073	3.5660	0.0065**
Arousal×FC	0.0245	0.0076	3.2260	0.0213*
Arousal×P	0.0215	0.0073	2.9650	0.0484*

Dependent variable = task offset				
Arousal×F	0.0310	0.0078	3.9980	0.0011**
Arousal×FC	0.0244	0.0079	3.0900	0.0261*
Arousal×P	0.0268	0.0076	3.5500	0.0062**
Arousal×PO	0.0364	0.0078	4.6840	<0.001***
Arousal×T8	-0.0242	0.0075	-3.2060	0.0188*
Valence×F	0.0246	0.0076	3.2460	0.0175*
Valence×PO	0.0450	0.0076	5.9490	<0.001***
Valence×T7	-0.0223	0.0075	-2.9680	0.0360*

Dependent variable = task alpha power				
Arousal×PO	-0.0168	0.0051	-3.3090	0.0197*

The stars indicate significant values, with the following thresholds: *p < 0.05 (range: 0.01–0.05), **p < 0.01 (range: 0.001–0.01), ***p < 0.001 (range: 0–0.001).

### 3.3 Spectral modulations with enhanced inhibition of relay population by reticular population or other direct cortical feedback

A corticothalamic model was set up to probe the mechanisms underlying changes in periodic and aperiodic properties of ongoing EEG activity with emotional stimuli (Fig. 2). In the absence of stimuli, the population dynamics in relay nuclei is driven by noisy inputs, and the resulting power spectra correspond to a resting awake state (Fig. 2). Thus, from the dynamical systems point of view, the resultant brain dynamics emerging from corticothalamic interactions is akin to a damped nonlinear oscillator with a natural frequency lying in the alpha range (8–12 Hz).

**Fig. 4.**
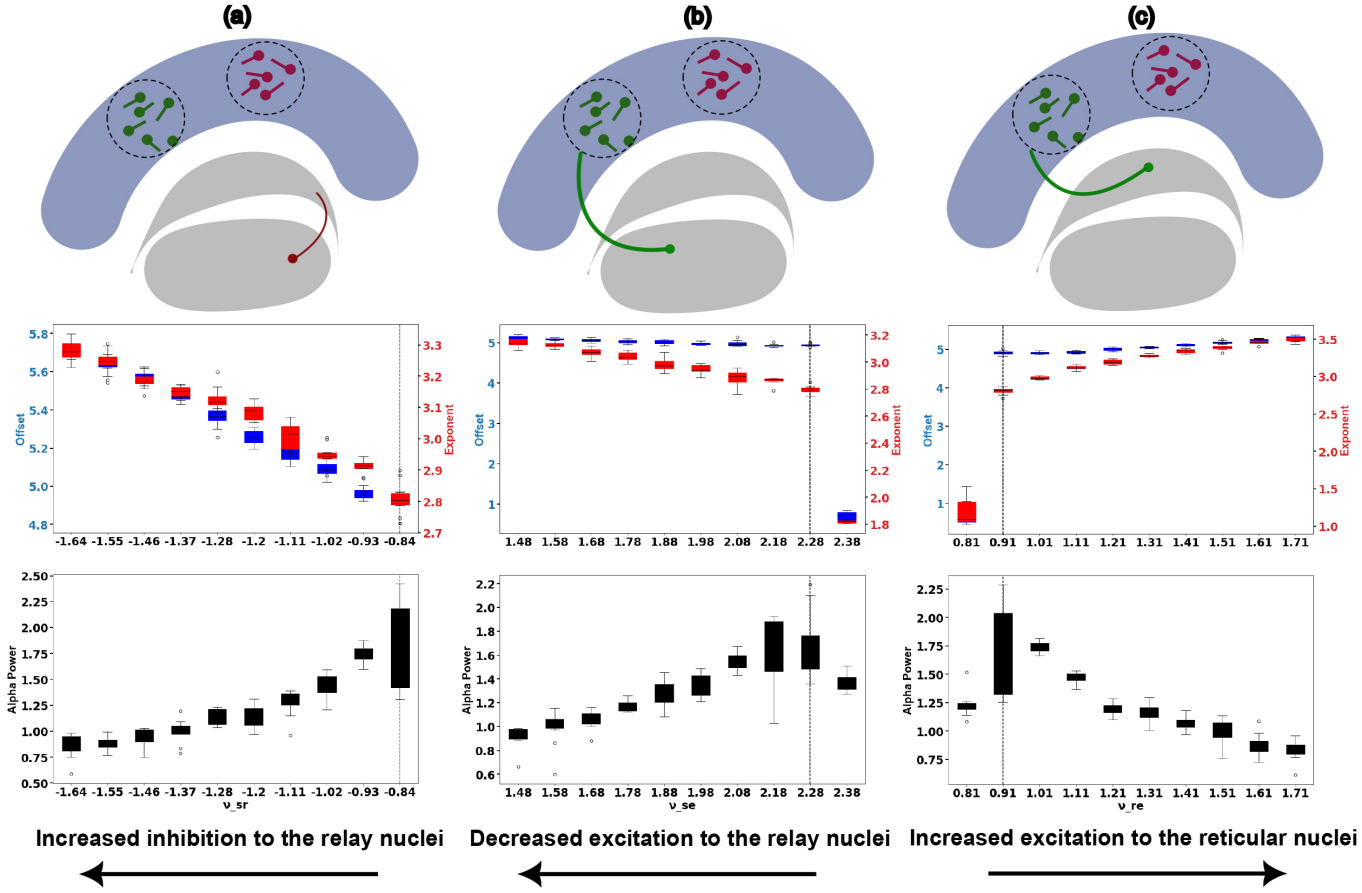
Offset (blue), Exponent (red), and Alpha Power (black) of simulated power spectra as functions of varying connectivity topologies, specifically synaptic strength. Each configuration (a–c) displays the impact of adjusting a single synaptic connection, while all other connections are held constant as shown inFigure 2. Vertical dashed lines represent the normative values for each parameter, based on prior studies (Freyer et al., 2011;Robinson et al., 2001). This figure focuses on the parameter configurations that align with empirical observations, offering insight into how connectivity modulations influence spectral properties. Additional parameter variations are provided inSupplementary Figure S4for a comprehensive view of connectivity effects on spectral dynamics.

Since arousal showed significant effects along exponent, offset, and alpha peak power, we simulated the brain dynamics for different topological configurations of corticothalamic connectivity (Fig. 4a–c,Supplementary Fig. S4a–e). Subsequently, we sought to identify the plausible configurations for generating the identified pattern of variation of alpha power decrease and increase in aperiodic components. Numerical integration ofEquation (6)with the Euler method and step size of 1 ms was performed. Transients were removed from the simulated time series, following which the resulting simulated spectra were modeled with the FOOOF algorithm (same parameters as the empirical EEG analysis) to extract periodic and aperiodic features, namely the peak alpha power and the offset and exponent of the background activity.

Our primary candidate for the arousal mechanism was the interaction between the thalamic reticular nuclei and the relay nuclei (Zikopoulos & Barbas, 2012). We also explored the possibility that modulations in other thalamic and cortical connections could represent alternative pathways contributing to arousal. To that end, we performed simulations by varying each coupling parameter around its normative values while keeping all other parameters fixed and noting how this altered spectral features (exponent, offset, and alpha power) of the resulting signal (Fig. 4a–c,Supplementary Fig. S4a–e). This exhaustive approach revealed increased inhibition to relay nuclei as the strongest candidate to account for observed alterations in spectral features as a consequence of arousal (Fig. 4a). WhileFigure 4adisplays the scenario when relay nucleus is inhibited from reticular nuclei directly,Figure 4b and crepresents the scenarios when inhibition to relay is effectuated indirectly through the direct decrease in excitation to relay from cortex and increased excitation to reticular nucleus which in turn amplifies reticular to relay inhibition, respectively. We observed an increased 1/f slope (exponent) and offset slope (in absolute values) and decrease in alpha peak power, similar to the EEG empirical results. These changes occurred when the relay nuclei received increased inhibitory input, or reduced excitatory input (Fig. 4).

## 4 Discussion

The primary aim of our study was to investigate how emotional states in a naturalistic setting conceptualized along dimensions of arousal and valence are represented in EEG signals. In this context, we observed that the role of periodic and aperiodic background activity in relation to emotional dimensions has been underexplored. With the current understanding in the field that both rhythmic and arrhythmic activities contribute toward cognitive and physiological processing (Donoghue et al., 2020;Voytek et al., 2015), we hypothesized that arousal and valence may be differentially represented along these signal attributes. Our findings demonstrated a novel significant increase with arousal in both parameters that define the aperiodic brain activity, namely, exponent (slope in power spectrum) and offset (y-intercept in the power spectrum) during the viewing of video clips perceived as augmented high arousal. This result suggests a potential connection between the aperiodic components and the intensity of emotional arousal, expanding our understanding beyond the commonly studied rhythmic components. We did not observe a global effect of aperiodic components with valence experience, suggesting that the influence of valence on these EEG features may be subtler. The decrease in peak alpha power with high arousal induction aligns with previous studies linking alpha power to emotional arousal (Hofmann et al., 2021;Kim et al., 2021;Schubring & Schupp, 2021). Digging deeper to uncover the mechanistic underpinnings of the arrhythmic and rhythmic constituents, we utilized the corticothalamic model of macroscopic EEG waves established byRobinson et al. (1997,^2003^) in our investigation. By exploring different possible circuit mechanisms, we could identify that increased inhibition to relay nuclei within this model can replicate the pattern of changes in aperiodic and periodic activity with increasing arousal. Based on the findings ofZikopoulos and Barbas (2012)andJohn et al. (2016), we hypothesized that increased coupling from the reticular to relay nuclei is the mechanism behind emotional arousal processing. However, we also found that feedback loops from the cortex to the reticular and relay nuclei can explain our results. Interestingly, despite considering these three possible corticothalamic interactions involving top-down and bottom-up influences, the ultimate mechanism remains inhibitory, supporting the concept of inhibitory gating of stimuli resulting from emotional arousal, which we further elaborate on in the follow-up section.

### 4.1 Representation of inhibition in neural oscillations

Following several prior research studies, the reduction in alpha peak power is observed during high arousal states (Hofmann et al., 2021;Kim et al., 2021;Schubring & Schupp, 2021). This decline in alpha power has been proposed to result from heightened attentional demands (Ghosh et al., 2021;Hofmann et al., 2021;Jensen & Mazaheri, 2010;Kim et al., 2021;Schubring & Schupp, 2021;Weisz et al., 2011;Wöstmann et al., 2021). Gating by inhibition proposes that in task-irrelevant area, alpha increases to exhibit active inhibition. Thus, decrease in alpha at parietooccipital areas and frontal areas (not significant) could imply functional coupling for attentional orientation (Misselhorn et al., 2019).

The global exponent of 1/f component exhibits a positive relation with arousal. This exponent is the negative counterpart of the slope within the equations utilized by our algorithm. In the context of aperiodic spectra, the slope has been proposed to represent a balance between excitation and inhibition, as demonstrated in previous studies (Gao et al., 2017;Miller et al., 2009;Voytek et al., 2015). The power spectral density (PSD) exponent characterizes decaying power law of frequencies. Studies have suggested with higher inhibition, there is more representation of slow GABA-mediated effect (e.g.Gao et al., 2017). These slow inhibitory synaptic currents support depolarization timings of slower frequencies and not sustaining the faster frequencies. Consequently, an increase in the exponent may indicate increased inhibition. A steeper slope (more negative) or a larger exponent, therefore, suggests a greater degree of inhibition relative to excitation. Our results consistently indicate a higher exponent with increased arousal elicitation, signifying enhanced inhibitory processes during heightened emotional states. This observation suggests that high emotional arousal may involve active inhibitory control, marking a departure from certain studies that have linked arousal, defined in terms of vigilance levels, for example, command following, eyes opening (Boly et al., 2013) during conscious states of sleep, anesthesia, and wakefulness to the 1/f slope (Colombo et al., 2019;Lendner et al., 2020). Notably, the slope was found to be more negative during sleep stages compared with the awake state. It is important to emphasize that different arousal types are defined in the neuroscience literature; sleep–wake, alertness level, physiological, sexual, and emotional (Hofmann et al., 2021;Pfaff et al., 2012). Arousal in the context of sleep studies typically pertains to the nonspecific activation of the cortex within the sleep–wake dimension (Oken et al., 2006). Nevertheless, they are different processes and yet may not be completely dissociable, they share some psychological (e.g. enhanced sensorimotor activation, emotional reactivity) and physiological (e.g. sympathetic activations) characteristics (Hofmann et al., 2021;Kreibig, 2010;Pfaff et al., 2012). On this account, the opposite finding of the exponent observed in our study suggests that the mechanism of arousal in emotional contexts may differ from arousal within the sleep–wake dimension.

The aperiodic offset can give a qualitative description of average firing rate of neuronal populations (Manning et al., 2009;Miller et al., 2009) and can be conceptualized as an indicator of the overall neural activity or cortical excitability (Weber et al., 2020).Miller et al. (2009)proposed that spectral offset reflects broadband power associated with overall neuronal activity. In ECoG data from finger movement tasks, they observed an increased 1/f offset during active states, indicating uniform power increase across frequencies without changes in slope. A Poisson-based neuron model confirmed that higher input rates raised power spectral density (PSD) amplitude while maintaining slope. Similarly,Manning et al. (2009)found that broadband power shifts correlated with increased firing rates, showing broadband power as a more reliable predictor of spiking than narrowband oscillations.Weber et al. (2020)further linked broadband power reduction to lower neural activity during a 120-day isolation period. Together, these findings suggest spectral offset as an indicator of overall neuronal activity rather than specific rhythms. Consequently, this interpretation implies an elevated level of cortical activation during high arousal in comparison with low arousal settings. At cluster level, we found significant increase in offset in frontal, frontocentral, parietal, and parietooccipital while decrease in right temporal with arousal increase. We also observed significant increase of offset with valence at parietooccipital and frontal while decrease in left temporal site. It might be more insightful to interpret these findings in combination rather than single components, as it will become clear in subsequent text.

### 4.2 A plausible mechanism of emotion attention modulation at thalamic level

In light of the primate studies demonstrating direct connections between the amygdala and the thalamic reticular nucleus, particularly in the context of emotion and selective attention (Barbas et al., 2011;Zikopoulos & Barbas, 2012) and the role of the thalamic reticular nucleus in sensory processing, as shown in studies involving rats (Ahrens et al., 2013;Aizenberg et al., 2019;Shosaku, 1986), we sought to translate these findings along with our observations into a computational model of the corticothalamic system. Moreover, the (John et al., 2016) model shows how the amygdala–thalamic reticular circuit can mediate both top-down and bottom-up attentional control, influenced by emotional salience. The model suggests that this pathway enables the selective suppression of irrelevant stimuli and the enhancement of emotionally relevant information before reaching cortical processing centers. This computational mechanism aligns with the primate-based anatomical findings fromBarbas et al. (2011)andZikopoulos & Barbas (2012)which demonstrate similar connectivity patterns, in the primate brain. This provides a theoretical framework that emotion-guided selective attention is achieved through inhibition by the thalamic reticular nuclei.

Building on this foundation of thalamic reticular nuclei, our model introduces a framework that explores the oscillatory dynamics within the corticothalamic system under varying states of emotion arousal. Our model receives noisy ascending inputs and behaves akin to a damped oscillator, returning to baseline activity following perturbations at a characteristic frequency. The model was designed to receive noisy ascending inputs and behaves akin to a damped oscillator, returning to baseline activity following perturbations at a characteristic frequency. In our model, we use the global mean membrane voltage of excitatory cortical populations to represent cortical activity. Importantly, the resting dynamics of our model encompasses the key variables of interest in our study, including a prominent spectral peak in the alpha band and distinctive 1/f background activity, as previously observed in empirical studies (Robinson et al., 1997,^2003^). Modulating the inhibitory coupling strength from the reticular corticothalamic unit allowed us to explore how the spectral statistics of the alpha band and background aperiodic activity align with empirical observations. We further suggest that such inhibitory influences on the relay nuclei may plausibly arise from an amygdala-to-thalamic reticular connection (John et al., 2016;Zikopoulos & Barbas, 2012), though this remains speculative and was not directly tested within the current framework.

Inhibition from thalamic reticular nuclei to relay nuclei is through both GABA-A (fast inhibitory time scale) and GABA-B (slow inhibitory time scale) synapses. Thalamic reticular nuclei have been shown to exhibit spindle oscillations (9–15 Hz) in cortex during sleep (e.g.Halassa & Acsády, 2016). Given the slow inhibitory action of GABA-B receptors (100–150 ms), these have been implicated in the alpha rhythm range. Although our model does not explicitly differentiate between these types of inhibition, the decrease in alpha power observed could be indicative of either reduced corticothalamic excitatory activity or disrupted synchrony. Enhanced thalamic reticular nuclei inhibition may reduce the activity of thalamic relay neurons, leading to a drop in alpha power. Additionally, increased thalamic reticular activity could disrupt synchrony between corticothalamic and cortical neurons, impairing the circuits responsible for generating alpha rhythms.

Interestingly, in humans, evidence of direct connections has been reported between amygdala and pulvinar thalamic nuclei (Abivardi & Bach, 2017;Tamietto et al., 2012) and also been suggested as a pathway for emotional attention (Pessoa & Adolphs, 2010;Vuilleumier, 2005). With respect to this, another possible modulation can be from amygdala to relay nucleus which will have to be inhibitory in nature. This requires, however, a detailed investigation in future studies with direct anatomical measurements currently out of scope of the present dataset. We also observe that decreased coupling strength from cortex to relay nucleus and increased coupling strength from cortex to reticular nucleus can also give rise to empirical observations. This suggests that although the inhibition of relay nuclei is an important criterion for aperiodic and periodic oscillatory features linked to arousal, the routes to achieve this may be multiple. Thus, alternative to amygdala–reticular functional coupling, decrease in cortical excitation to relay nuclei and in contrast an increase of corticoreticular excitation can also lead to a net effect of increased inhibition to relay nuclei.

### 4.3 Limitations and future directions

In examining emotional responses to videos, various factors impact the analysis methodology and its interpretation. Firstly, the study is constrained by assessing subjective emotions only after the video concludes. Thus, we assume that reported and felt emotional states remain constant throughout that 1 minute of video presentation. However, it is important to acknowledge that emotions may evolve during this period, and the fixed assessment may not fully capture potential changes. Consequently, this does not allow for temporal modeling of thalamic gating of emotion. Additionally, the model assumes cortex as an interaction of a single excitatory and inhibitory population, lacking consideration for spatial specificity. This limits understanding of the spatiotemporal dynamics of emotional experiences during video exposure. To comprehensively evaluate the results, it is crucial to interpret them within the context of continuous rating and on a subject-specific basis. Thus, it is essential to recognize that individuals experience emotions uniquely and that emotional responses can fluctuate over time (Hofmann et al., 2021). Lastly, the various excitation and inhibition scenarios among the relevant nodes, cortical ensembles, reticular, and relay nuclei all leading to an increased net inhibition to relay nuclei need to be verified in anatomical studies from animal models. Lastly, it is possible to perform a detailed model fit to extract relevant parameters of the model using simulation-based inference algorithms. Indeed, since the model has also been solved in the Fourier domain (Robinson et al., 2001), it may even be possible to directly fit the empirical power spectra in the frequency space itself. This would be our goal for future studies where we want to explore further use cases of the model in understanding oscillatory activity during tasks where the effective connectivity profiles in consideration are guided by a specific hypothesis.

## Ethics

The DEAP dataset team reviewed and approved the studies involving human participants. All participants provided their written informed consent to be part of this study.

## Supplementary Material

Supplementary Material

## Data Availability

DEAP data used in this paper can be downloaded athttps://www.eecs.qmul.ac.uk/mmv/datasets/deap/. The FOOOF analysis pipeline can be downloaded athttps://github.com/fooof-tools/fooof_mat. The Python codes for running simulations of the corticothalamic model can be downloaded fromhttps://bitbucket.org/cbdl/corticothalamicmodelofemotion/src/main/CorticoThalamicModel/ A separate Python notebook which involves all functions of the repository is provided as aSupplementary file.
