# Supplementary material for: Inhibition of thalamic relay nuclei scales the aperiodic and alpha band oscillations associated with arousal during naturalistic stimulus viewing
Source: Imaging Neurosci (Camb). 2025 Jan 23;3:imag_a_00451. doi: 10.1162/imag_a_00451 (PMC12319808; doi:10.1162/imag_a_00451)
Supplement: Supplementary Material [file imag_a_00451-supp.zip › imag_a_00451-supp.pdf]

Supplementary material for:

Inhibition of thalamic relay nuclei scales the aperiodic and alpha band oscillations associated with arousal during naturalistic stimulus viewing

Authors: Ritu M Borah, Anagh Pathak, Arpan Banerjee

The three levels of corticothalamic interactions (Robinson et al., 1997, 2003)

$$\frac{1}{\alpha\beta}\frac{\partial^2 V_e}{\partial t^2} + \left(\frac{1}{\alpha} + \frac{1}{\beta}\right)\frac{\partial V_e}{\partial t} + 1 = \nu_{ee}\phi_e(t) + \nu_{ei}\phi_i(t) + \nu_{es}\phi_s(t - t_0/2) \ldots. (s\ 1)$$

$$\frac{1}{\alpha\beta}\frac{\partial^2 V_r}{\partial t^2} + \left(\frac{1}{\alpha} + \frac{1}{\beta}\right)\frac{\partial V_r}{\partial t} + 1 = \nu_{\mathfrak{R}}\phi_e(t - t_0/2) + \nu_{rs}\phi_s(t) \ldots (s2)$$

$$\frac{1}{\alpha\beta}\frac{\partial^2 V_s}{\partial t^2} + \left(\frac{1}{\alpha} + \frac{1}{\beta}\right)\frac{\partial V_s}{\partial t} + 1 = \nu_{se}\phi_e(t - t_0/2) + \nu_{sr}\phi_r(t) + \nu_{sn}\phi_n(t) \ldots (s3)$$

Equations for solving the three levels of corticothalamic interactions, corresponding to the dynamics presented in Figure 2 and 3 of the main text. These equations describe the interactions within the corticothalamic system by capturing the evolution of membrane potentials V in excitatory (V_e), reticular (V_r), and specific/relay (V_s) populations as they interact over time t.

Equation (S1) describes the dynamics of the excitatory population, where V_e is influenced by self-coupling ν_{ee}, input from inhibitory populations ν_{ei}, and thalamic relay input ν_{es} with a time delay $t_0/2$.

Equation (S2) governs the reticular population V_r, incorporating excitatory input $\nu_{\mathfrak{R}}$,with a delay $t_0/2$ and input from relay nucleus ν_{rs}.

Equation (S3) models the relay population V_s, driven by excitatory input ν_{se},with a delay $t_0/2$, input from reticular ν_{sr}, and non-specific sensory input ν_{sn}.

Self Assessment Manikin for affective dimensions

To report the ratings across affective dimensions, Self Assessment Manikin was used. Each SAM scale allows participants to visually rate their emotional responses on a continuum, with Valence assessing the positive to negative emotional range, Arousal measuring activation level from calm to excited, Dominance evaluating the sense of control or influence, and Liking reflecting the degree of favorability. The SAM scales provide an intuitive method for self-reported ratings, facilitating consistent subjective assessments across affective dimensions. (Koelstra et al., 2012)

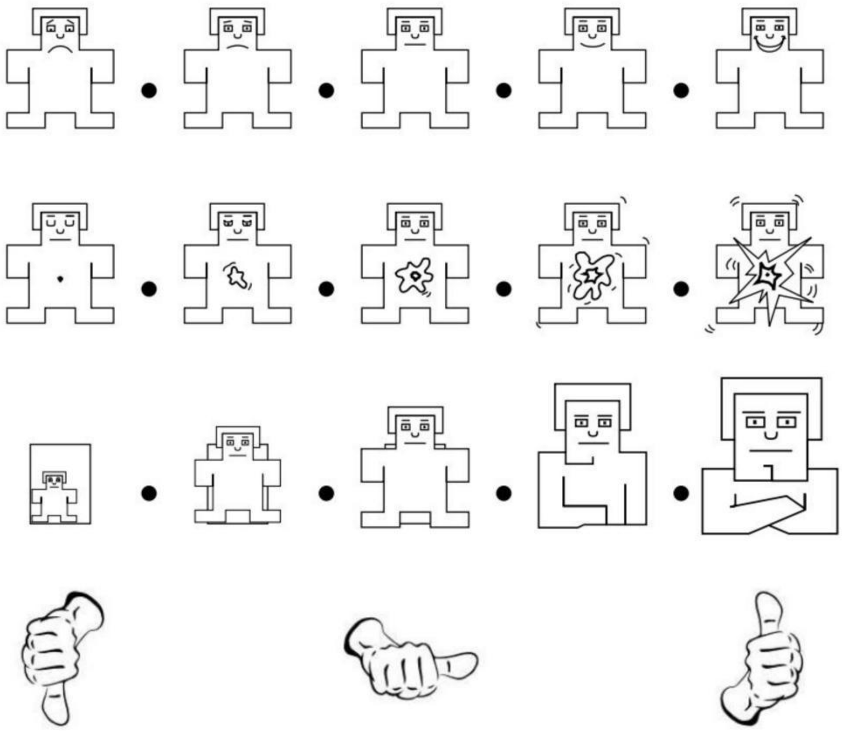

Figure S1:*Self-Assessment Manikin (SAM) scales used for participant ratings across affective dimensions. From top to bottom: Valence SAM, Arousal SAM, Dominance SAM, and Liking.*

Power Spectrum of empirical data

To analyze the key spectral features of interest—specifically, the alpha peak power, the 1/f slope, and the spectral offset—the Fitting Oscillations and One-Over-F (FOOOF) algorithm was applied to each subject's power spectrum. This approach allowed for a decomposition of the power spectrum into distinct components, separating periodic (oscillatory) features from the aperiodic (1/f) background activity. Figure S2 presents the spectral profiles of selected subjects, illustrating individual differences in alpha peak detection by the FOOOF model.

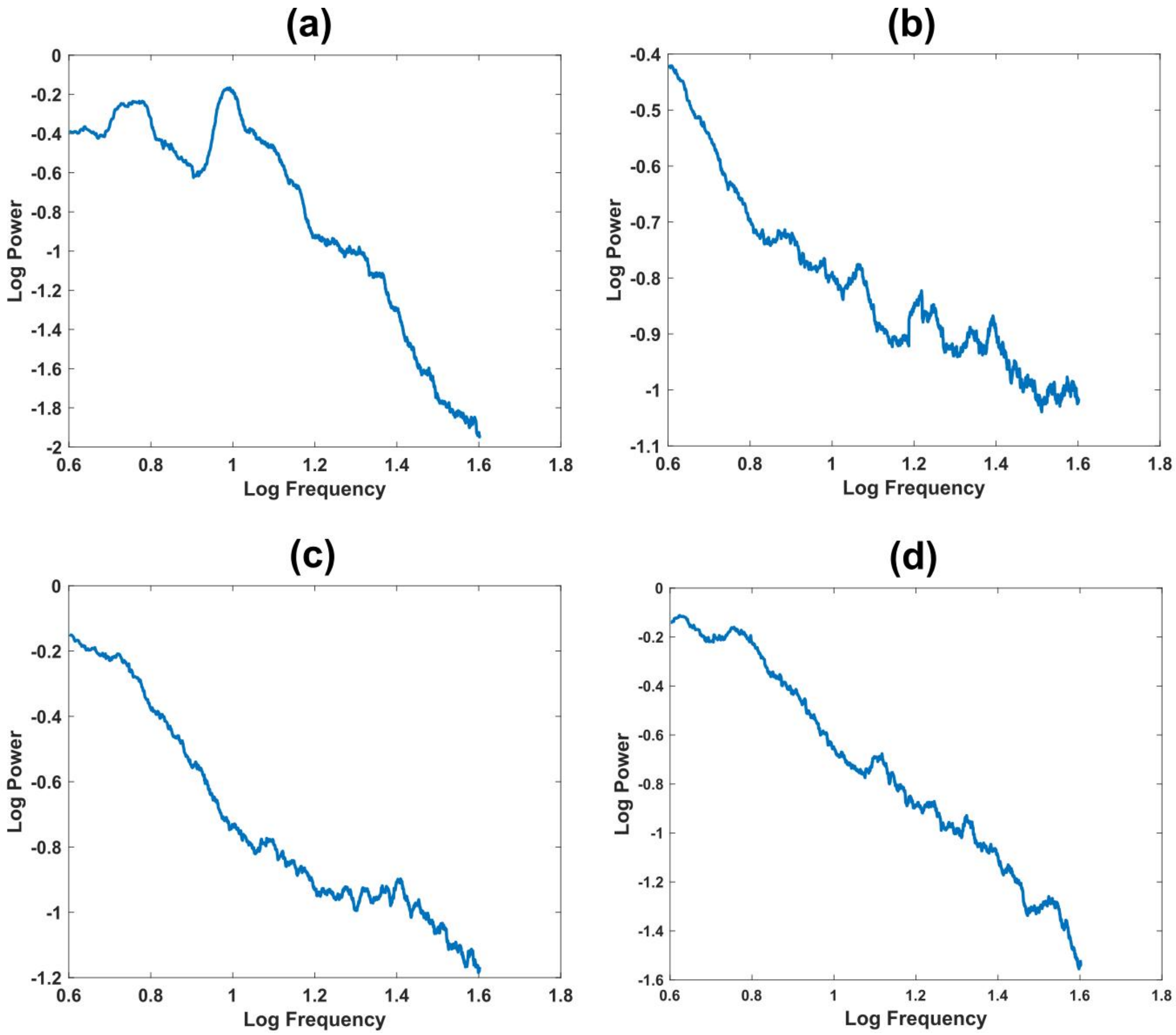

Figure S2: *Spectral representation of individual trial data for selected subjects, illustrating variations in alpha peak detection by the FOOOF model. Panel (a) displays the spectrum for Subject 1, where an alpha peak was successfully identified by the model. Panels (b), (c), and (d) show spectra from trials of Subject 11, Subject 15, and Subject 27, respectively, where the model did not detect an alpha peak. This figure highlights individual differences in spectral profiles and demonstrates instances where the FOOOF model's peak detection criteria did not capture an alpha peak.*

Spectral features at high—low arousal and valence responses

To demonstrate the spectral differences between the arousal and valence, a group level analysis segregation was performed. The spectrum was derived by averaging across participants for each condition, with a threshold rating of 5 distinguishing high and low values (Koelstra et al., 2012). Here, subjective variability in individual ratings is not considered, emphasizing the general trend across participants rather than individual differences. Spectral characteristics were averaged separately within high and low groups for both arousal and valence to visualize overarching patterns associated with these emotional dimensions.

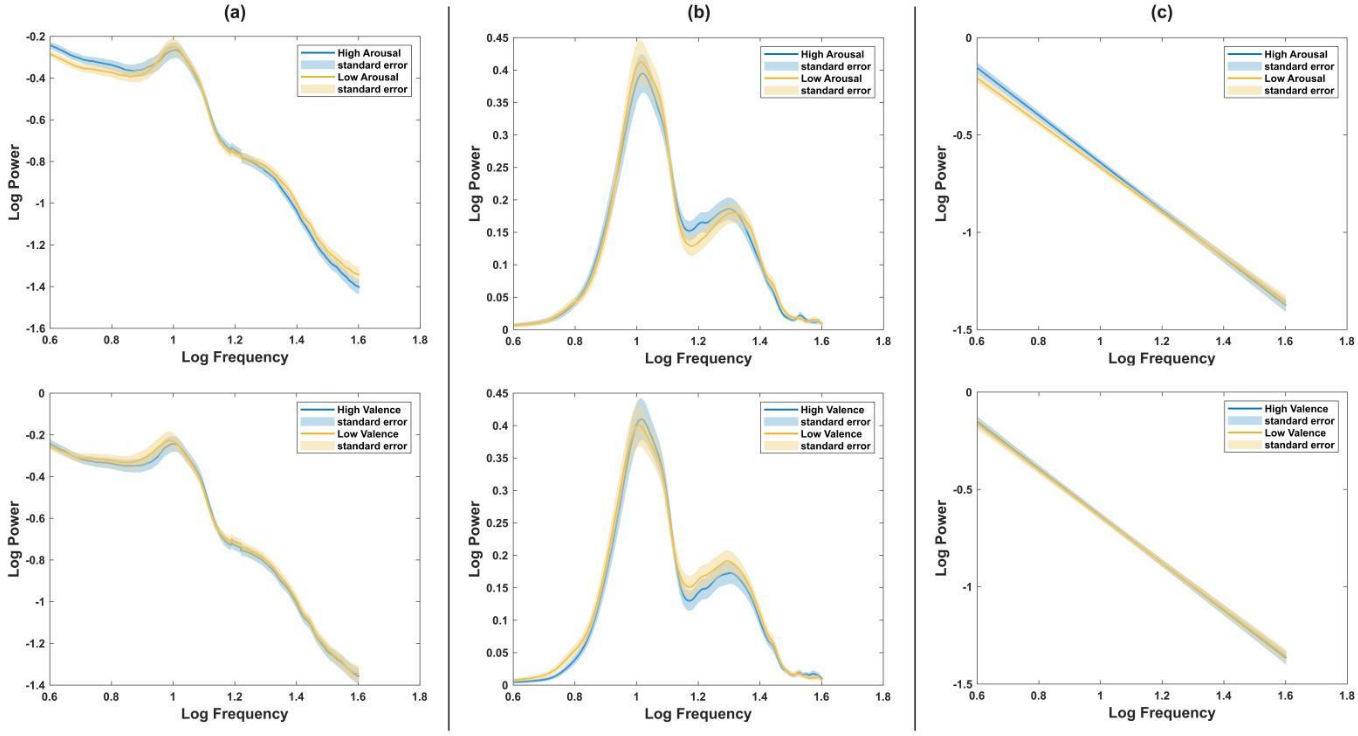

Figure S3: *Group-level spectrum distribution across high and low arousal and valence ratings.*

Spectral dynamics driven by corticothalamic modulation of synaptic strengths

We performed a comprehensive parameter sweep of all possible synaptic coupling parameters to demonstrate that the coupling between the thalamic reticular nucleus (TRN) and the thalamic relay nucleus (STN) is the best theoretical candidate for the arousal mechanism. The other parameters we examined included coupling strengths between: (1) cortex to STN, (2) cortex to TRN, (3) thalamic relay nuclei to cortex, (4) cortex to cortex, (5) STN to TRN, (6) inhibitory cortical populations to excitatory cortical populations, and (7) sensory afferents to STN. We have reported the trends here in Figure S4.

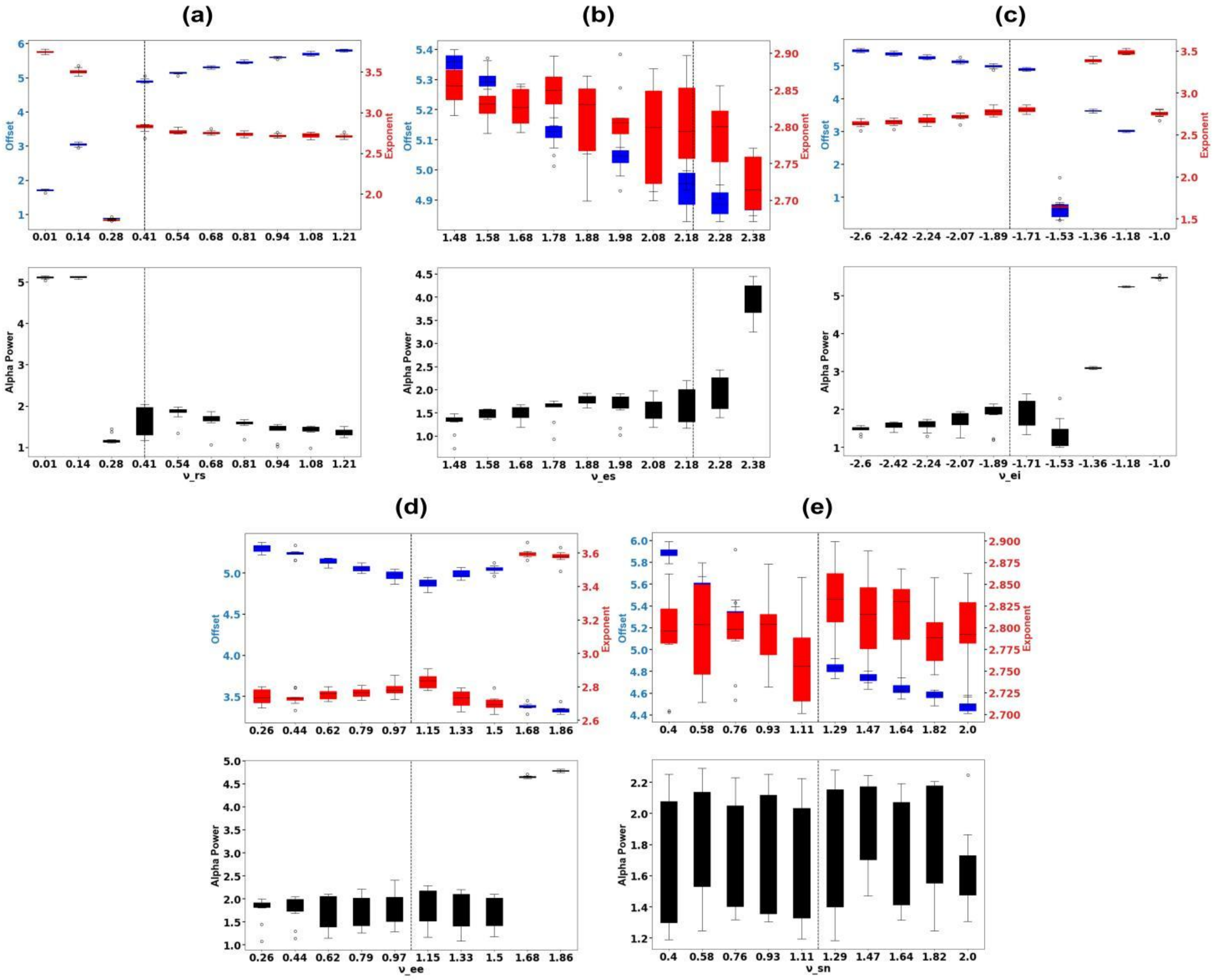

Figure S4: *Examination of variability in neural dynamics with changes in synaptic strength across their normative values within the thalamocortical model. This supplementary figure extends the main text findings(Results Section 3.3) by illustrating the effects of varying synaptic strengths in additional pathways beyond those covered in Figure4 (a)–(c) in the main text. Here, synaptic strengths were systematically adjusted within normative biological ranges to explore their impact on aperiodic and periodic components of spectral power. The additional pathways shown in this figure further elucidate the robustness and sensitivity of thalamocortical dynamics across multiple connection types, offering a broader understanding of the system's response to physiological synaptic changes.*

Linear mixed effect results

All model estimates are included here, whereas only significant effects were reported in the main text. Holm-adjusted p-values are applied to control for multiple comparisons, ensuring robust statistical conclusions. This supplementary table provides a full account of the interaction effects across clusters, offering detailed insight into each interaction term's role in explaining the variance in the model.

Table S1:This table presents the comprehensive results of the mixed model analysis described by the equation(5) in the main text.

Factors	β	Standard error	Z value	p-value
		Dependent variable=Task Exponent		
**Pre-stimulus Exponent×C**	0.3175	0.0164	19.3450	<0.001***
**Pre-stimulus Exponent×CP**	0.2703	0.0157	17.2630	<0.001***
**Pre-stimulus Exponent×F**	0.2937	0.0170	17.2780	<0.001***
**Pre-stimulus Exponent×FC**	0.3578	0.0169	21.1990	<0.001***
**Pre-stimulus Exponent ×P**	0.2638	0.0162	16.2900	<0.001***
**Pre-stimulus Exponent×PF**	0.2696	0.0175	15.3890	<0.001***
**Pre-stimulus Exponent×PO**	0.3577	0.0172	20.8130	<0.001***
**Pre-stimulus Exponent×T7**	0.3095	0.0167	18.5260	<0.001***
**Pre-stimulus Exponent×T8**	0.3150	0.0174	18.0780	<0.001***
**Arousal×C**	0.0345	0.0074	4.6330	<0.001***
**Arousal×CP**	0.0260	0.0073	3.5660	0.0065**
Arousal×F	0.0196	0.0074	2.6300	0.1279
**Arousal×FC**	0.0245	0.0076	3.2260	0.0213*
**Arousal×P**	0.0215	0.0073	2.9650	0.0484*
Arousal×PF	0.0094	0.0074	1.2660	1.0000
Arousal×PO	0.0084	0.0074	1.1410	1.0000
Arousal×T7	-0.0042	0.0073	-0.5700	1.0000
Arousal×T8	-0.0059	0.0073	-0.8060	1.0000
Valence×C	-0.0076	0.0072	-1.0570	1.0000
Valence×CP	-0.0019	0.0071	-0.2650	1.0000
Valence×F	0.0103	0.0073	1.4210	1.0000
Valence×FC	-0.0074	0.0076	-0.9780	1.0000
Valence×P	0.0119	0.0072	1.6700	1.0000
Valence×PF	0.0103	0.0073	1.4080	1.0000
Valence×PO	0.0150	0.0072	2.0700	0.4612

Valence×T7	-0.0152	0.0072	-2.1060	0.4574
Valence×T8	-0.0040	0.0072	-0.5480	1.0000
Valence×Arousal	-0.0019	0.0008	-2.2750	0.3207
		Dependent variable = Task Offset		
**Pre-stimulus Offset×C**	0.3311	0.0169	19.5790	<0.001***
**Pre-stimulus Offset×CP**	0.2703	0.0154	17.5930	<0.001***
**Pre-stimulus Offset×F**	0.2998	0.0171	17.4980	<0.001***
**Pre-stimulus Offset×FC**	0.3254	0.0179	18.1760	<0.001***
**Pre-stimulus Offset ×P**	0.2653	0.0158	16.7700	<0.001***
**Pre-stimulus Offset×PF**	0.2893	0.0170	17.0220	<0.001***
**Pre-stimulus Offset×PO**	0.4065	0.0169	24.0940	<0.001***
**Pre-stimulus Offset×T7**	0.3255	0.0175	18.6390	<0.001***
**Pre-stimulus Offset×T8**	0.2777	0.0184	15.0700	<0.001***
Arousal×C	0.0204	0.0077	2.6670	0.0680
Arousal×CP	0.0119	0.0075	1.5800	0.4564
**Arousal×F**	0.0310	0.0078	3.9980	0.0011 **
**Arousal×FC**	0.0244	0.0079	3.0900	0.0261*
**Arousal×P**	0.0268	0.0076	3.5500	0.0062**
Arousal×PF	0.0219	0.0077	2.8260	0.0519
**Arousal×PO**	0.0364	0.0078	4.6840	<0.001***
Arousal×T7	-0.0187	0.0076	-2.4530	0.0993
**Arousal×T8**	-0.0242	0.0075	-3.2060	0.0188*
Valence×C	-0.0160	0.0075	-2.1290	0.1661
Valence×CP	-0.0089	0.0074	-1.2070	0.6819
**Valence×F**	0.0246	0.0076	3.2460	0.0175*
Valence×FC	0.0021	0.0079	0.2670	0.7898
Valence×P	0.0196	0.0074	2.6320	0.0690
Valence×PF	0.0209	0.0076	2.7540	0.0589
**Valence×PO**	0.0450	0.0076	5.9490	<0.001***
**Valence×T7**	-0.0223	0.0075	-2.9680	0.0360*
Valence×T8	-0.0080	0.0075	-1.0600	0.6819
Valence×Arousal	-0.0020	0.0009	-2.2770	0.1367
		Dependent variable = Task Alpha power		
Pre-stimulus Alpha×C	0.1107	0.0163	6.8040	<0.001***
Pre-stimulus Alpha×CP	0.1908	0.0132	14.5110	<0.001***

**Pre-stimulus Alpha×F**	0.0798	0.0150	5.3370	<0.001***
**Pre-stimulus Alpha×FC**	0.0739	0.0167	4.4220	0.0002***
**Pre-stimulus Alpha×P**	0.1447	0.0128	11.3260	<0.001***
**Pre-stimulus Alpha×PF**	0.0991	0.0146	6.7810	<0.001***
**Pre-stimulus Alpha×PO**	0.0689	0.0143	4.8220	<0.001***
Pre-stimulus Alpha×T7	-0.0162	0.0190	-0.8530	1.0000
Pre-stimulus Alpha×T8	-0.0259	0.0195	-1.3280	1.0000
Arousal×C	-0.0032	0.0051	-0.6240	1.0000
Arousal×CP	0.0097	0.0050	1.9360	1.0000
Arousal×F	-0.0042	0.0051	-0.8110	1.0000
Arousal×FC	-0.0078	0.0053	-1.4830	1.0000
Arousal×P	-0.0057	0.0050	-1.1430	1.0000
Arousal×PF	-0.0044	0.0051	-0.8680	1.0000
**Arousal×PO**	-0.0168	0.0051	-3.3090	0.0197*
Arousal×T7	0.0024	0.0051	0.4750	1.0000
Arousal×T8	0.0009	0.0051	0.1710	1.0000
Valence×C	0.0036	0.0054	0.6750	1.0000
Valence×CP	0.0043	0.0053	0.8050	1.0000
Valence×F	0.0029	0.0054	0.5280	1.0000
Valence×FC	-0.0075	0.0056	-1.3290	1.0000
Valence×P	-0.0015	0.0053	-0.2750	1.0000
Valence×PF	0.0034	0.0054	0.6270	1.0000
Valence×PO	-0.0116	0.0054	-2.1590	0.6176
Valence×T7	-0.0061	0.0054	-1.1230	1.0000
Valence×T8	-0.0012	0.0054	-0.2160	1.0000
Valence×Arousal	-0.0005	0.0006	-0.8450	1.0000
